# Laugh Headache, Not a Joke! A Case Report

**DOI:** 10.7759/cureus.22233

**Published:** 2022-02-15

**Authors:** Bob Daripa, Scott Lucchese

**Affiliations:** 1 Medicine, Grant Government Medical College and Sir J.J. Group of Hospitals, Mumbai, IND; 2 Internal Medicine: Neurology, Singapore General Hospital, Singapore, SGP; 3 Neurology, University Hospital Missouri, Columbia, USA; 4 Neurology, Headache, University of Arkansas for Medical Sciences, Little Rock, USA; 5 Neurology, Headache, University of Missouri School of Medicine, Columbia, USA

**Keywords:** cranio-spinal pressure dissociation, valsalva maneuver, tonsillar herniation, arnold-chairi malformation, laugh headache

## Abstract

Well delineated precipitating factors of migraine or incapacitating headaches are well known in the literature. Few peculiar and under-recognized precipitants are crying, shouting, straining in stools, urination, orgasm, childbirth, powerlifting. We present a case of a young student whose laughing aloud is a potent headache precipitant and is consistently reproducible despite normal brain imaging. It is worth mentioning here that laugh-induced headache has recently been assigned a place in the International Classification of Headache Disorders (ICHD-III) in 2018.

The proposed pathophysiology in our case could be loud laugh induced Valsalva maneuver raising intra-abdominal and intra-thoracic pressure momentarily causing venous congestion of head presenting as episodic headache. Another plausible explanation related to craniospinal pressure dissociation and the concept of dural elasticity and compliance needs to be explored if the symptoms persist and repeat scans show no pathology. Momentarily rise of intracranial pressure due to vigorous laugh could press the tonsils or distal cerebellar portion to herniate down transiently, causing symptoms and may be back to normal position once the laugh ceases. Social laughter releases enormous endogenous opioids, which is supported using positron emission tomography (PET) and u-opioid-receptor (MOR)-specific ligand carfentanil. A mirthful laugh could trigger a primary laugh headache. The role of modulated opioidergic activity and social mirthful laugh, if connected with such rare headaches requires further study.

## Introduction

Once, Hindu deity Lord Krishna experienced excruciating headaches, and when he asked Rishi raj, Narad Muni, a god sage, to bring dust from his devotee’s feet as a remedy. As per Hindu mythology, this well-known incident occurred in Dvapara Yuga (864,000 years back) [1]. Even in Greek mythology, Zeus (the god of sky and thunder), on experiencing intolerable headaches, ordered Hephaestus (God of blacksmiths) to crack open his skull with an axe [2]. Headache has an old rich history.

India, home to 16% of the world’s inhabitants, with a population of over 1.2 billion people, has a great impact on global headache demographic statistics [3]. A recent study from Bengaluru city (southern part of India) showed 63.9% headache prevalence in the young age group of 18-25 years [3,4], while the mean global prevalence stands at 14.7% [3]. Even the global burden of years living with a disability is very high with headaches where isolate migraine alone claims a second rank [3].

Well delineated precipitating factors of migraine or incapacitating headaches are well known in the literature. Few peculiar and under-recognized precipitants are crying, shouting, straining in stools, urination, orgasm, childbirth, powerlifting [5]. On the contrary, emesis raises intra-abdominal and intra-thoracic pressure, but it gives partial relief from migraine headache intensity, which could be due to increased arginine-vasopressin release [5].

We present a case of a young student whose laughing aloud is a potent headache precipitant and is consistently reproducible. It is worth mentioning here that laugh-induced headache has recently been assigned a place in the International Classification of Headache Disorders (ICHD-III) in 2018.

## Case presentation

A 19-year-old male engineering college student in good health (BMI 28) with no past medical, surgical, or illicit drug history presented in the clinic with a complaint of headache. It started around a year back, always event-related, and the last episode was 10 days back when he was attending a friends’ get-together. The patient observed that all these headaches precipitate acutely by a loud laugh, and while laughing, the headache builds up when the head naturally tilts back in a cervical extension position. It starts at the occipital-cervical region and spreads in a few seconds to involve the bilateral occipital region with a sense of grip accompanied by transient throbs lasting a few seconds or maybe a minute. The patient claims that he needs to suddenly quit his laugh at the peak of the laughing session as he senses some localized discomfort at the occipital region, which is not associated with any visual changes, giddiness, nausea, near fainting spells, tendency to fall, or any other bodily sensory or motor deficits. He tries to isolate himself from that humorous environment and sits/rests for a while, waiting for this headache to resolve on its own within a few minutes, and then he is back to normal without any residual effects.

The headache attacks were consistent with vigorous laughs and not related to Valsalva-like maneuvers or isolated neck bending or changing bodily position. The attacks were not reproducible if the intensity of the laugh was mild, and he claimed that humor inducted vigorous laugh serves as a good trigger for him. There were no associated symptoms suggesting migraine, cluster, or neuralgiform-type headache. He denies any continuous background headache or any form of ataxia. Also, there is no swallowing or chewing discomfort. Family members denied any chronic bothersome headaches or any other form of neurological disorders. The patient spends a good amount of time on his laptop (about 11-12 hours /day as work-related and mobile phone usage around 7-8 hours daily since the last two years), and he sleeps for about seven hours with no apnea or snoring.

**Figure 1:**
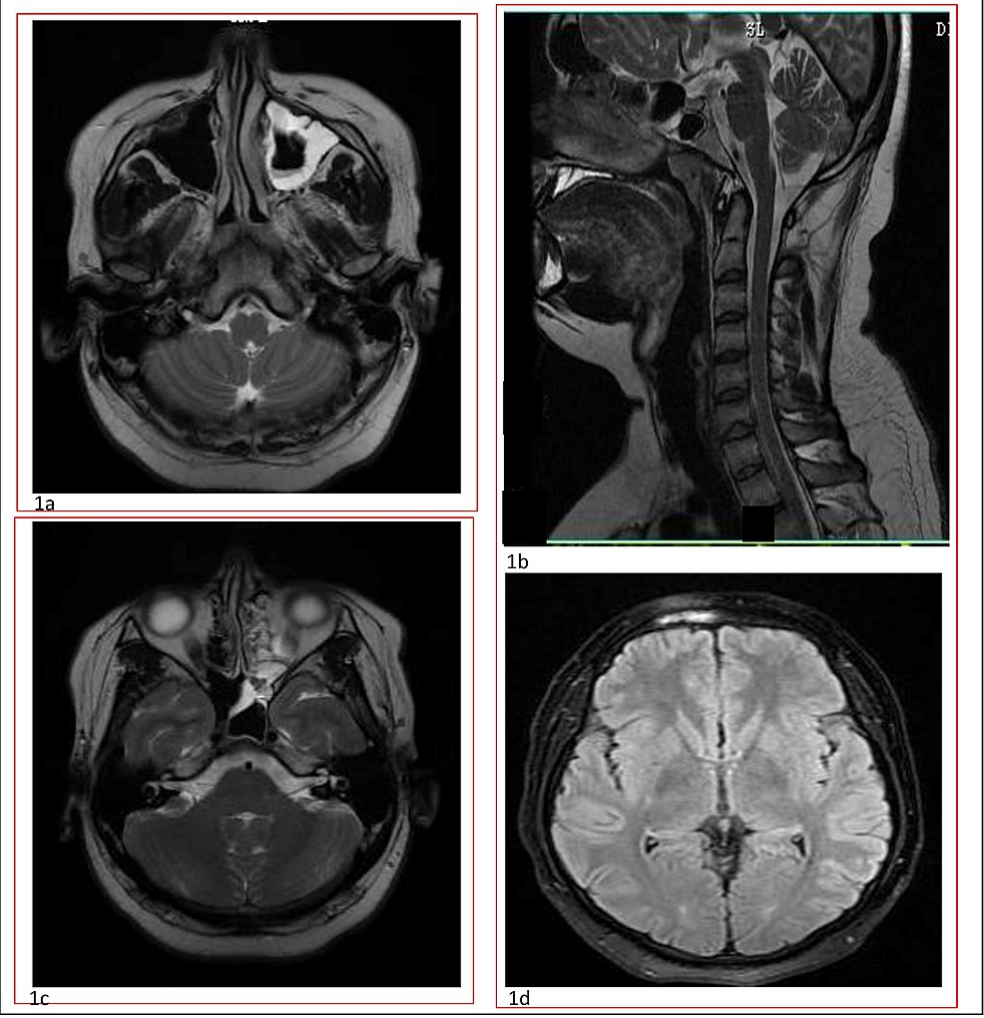
Axial section of T2 MRI brain at the level of the medulla (1a) and pons (1c) showing normal anatomy except deviated nasal septum. Figure (1b) is T2 MRI sagittal section showing the base of the brain where cerebellar tonsils are much above and odontoid process below McRae line indicating no herniation or basilar invagination or atlantoaxial impaction, Even Chamberlain line (line joining back of hard palate to opisthion) noted that tip of dens is below this line. Figure (1d) is the T2 Flair axial section of the brain at the level of the internal capsule and the basal ganglia showing normal anatomy.

Neurological examination was unremarkable with equal reactive pupils. Sensory examination was normal. No local tenderness in neck muscle with no manipulative maneuver provoked pain recorded neither any palpable trigger points identified. Blood investigation and EEG were within normal range. MRI brain with cervical spine screening along with chamberlain and ryes line are within normal limit with non-congested posterior fossa as shown in Figure 1. No medicines were offered at this point. There was no clinical change after six months of follow-up. The patient agreed to follow up in the clinic if notices any new symptoms or changes in current symptoms.

## Discussion

Very few cases of laugh-induced headache are reported so far [6,7], although, in clinical practice, the numbers might be more in specialized headache centers. Cases of a pathological laugh as in gelastic seizure, Angelman syndrome, or hypothalamic hamartoma, are well known [8]. Even laugh as a precipitant for cough headaches could also be seen [6]. In our case, no trigger points tenderness rules out spasmodic pain, and an MRI scan ruled out any bony deformity like platy basileus, atlantoaxial subluxation, disc bulge, or any other congenital anomaly. A similar case was reported in the past where the brain imaging was normal, which was categorized as laugh-induced headache [7].

The proposed pathophysiology in our case report could be loud laugh induced Valsalva maneuver raising intra-abdominal and intra-thoracic pressure momentarily causing venous congestion of head presenting as episodic headache. This could have also been accompanied by elevated episcleral and ocular choroidal bed venous pressure, which raises the intraocular pressure producing symptoms of blurring of vision or orbital pain, not present in our case [5].

Few cases were reported in the past where laugh-induced headaches were finally diagnosed with Arnold Chiari malformation type-1 based on MRI imaging findings, thus requiring surgical intervention. The associated signs symptoms of syringomyelia and syringobulbia with the above malformation could be present and should not be missed. Occasionally, tonsillar herniation or the dens of cervical vertebrae C1 could compress and may precipitate medullary signs [6].

Even isolated giant pacchionian arachnoid granulation presenting as severe laugh headaches are mentioned in literature [9]. This could ignite another plausible explanation related to craniospinal pressure dissociation [6] and the concept of dural elasticity and compliance, which needs to be explored if the symptoms persist and repeat scans shows no pathology. A rise of intracranial pressure due to vigorous laugh could press the tonsils or distal cerebellar portion to herniate down transiently, causing symptoms [6] but maybe back to normal position once the laugh ceases.

Social laughter releases enormous endogenous opioids, which were supported using positron emission tomography (PET) and u-opioid-receptor (MOR)-specific ligand carfentanil [10]. A mirthful laugh could trigger a primary laugh headache [7]. The connected role of modulated opioidergic activity and social mirthful laugh with such rare headaches is not known and requires further study.

## Conclusions

Headache is a much-neglected health condition in India that should be treated with grave seriousness and with a well-organized plan. Our case is unique as it needs to be handled with great care so that the impending emergencies can be anticipated well and can be prevented or taken care of. Be it of any variety, but headache-free people can be more productive with more earning potential spreading more awareness. The need is to train more professionals so that the understanding of headaches can be broadened.
